# The value of Angipoietin-2 as a biomarker for the prognosis of osteosarcoma: A protocol for systematic review and meta-analysis

**DOI:** 10.1097/MD.0000000000026923

**Published:** 2021-08-13

**Authors:** Lizhu Liu, Xinbo Zhang, Chaoyi Li, Ye Qu

**Affiliations:** a Department of Traumatic Surgery, The Second Affiliated Hospital of Hainan Medical College, Haikou, Hainan Province, China; b Department of Orthopaedic, The Second Affiliated Hospital of Hainan Medical College, Haikou, Hainan Province, China.

**Keywords:** Angipoietin-2, bioinformatics, meta-analysis, osteosarcoma, prognosis, protocol

## Abstract

**Background:**

The function of Angipoietin-2 (Agn2) in osteosarcoma has not been fully explored and exists controversial. Therefore, we conducted a meta-analysis to investigate the role of Agn2 in the prognosis of osteosarcoma. In addition, bioinformatics analysis was carried out to reveal the mechanism and related pathways of Agn2 in osteosarcoma.

**Methods:**

Literature search was operated on databases up to July 2021, including PubMed, Web of Science, China National Knowledge Infrastructure, China Biology Medicine disc, and Wan Fang Data. The relation between Agn2 expression and survival outcome was estimated by hazard ratio and 95% confidence interval. Meta-analysis was performed on the Stata 16.0. Being obtained from The Cancer Genome Atlas, the original data were used to further verify the prognostic role of Agn2 in osteosarcoma. Gene set enrichment analysis was applied to predict the potential mechanism of Agn2. The correlation between Agn2 and osteosarcoma immune infiltration was analyzed by TIMER database.

**Results:**

The results of this meta-analysis would be submitted to peer-reviewed journals for publication.

**Conclusion:**

This study will provide evidence for the exploration of the relationship between Agn2 and the prognosis of osteosarcoma and its mechanism.

**Ethics and dissemination:**

The private information from individuals will not be published. This systematic review also should not damage participants’ rights. Ethical approval is not available. The results will be published in a peer-reviewed journal or disseminated in relevant conferences.

**OSF registration number:**

DOI 10.17605/OSF.IO/GWQ53.

## 1 Introduction

Osteosarcoma refers to the most common bone malignancy in clinical practice, and it occurs mostly in adolescents, with high degree of malignancy. Accompanied by amputation and other serious consequences,^[1–3]^ lung metastasis can occur in the early stage. The 5-year survival rate with surgery alone is <20%.^[4]^ In recent years, with the progress of medical technology, especially the emergence of neoadjuvant chemotherapy combined with limb salvage surgery, the pain of amputation has been greatly reduced, but the 5-year survival rate of patients has also increased to about 80%.^[5,6]^ Despite this, about 30% of patients with osteosarcoma present with recurrence or lung metastasis during treatment or active treatment.^[7]^ Once lung metastases are diagnosed, the 5-year survival rate is only 20% to 30%.^[8]^ Therefore, it is very important to explore the mechanism of osteosarcoma metastasis, so as to evaluate the prognosis of patients.

Angiopoietin 2 (Ang2) is a secreted glycoprotein derived from adipose tissue and an important member of the angiopoietin like family.^[9–11]^ A number of studies have proved that, as a chronic inflammatory mediator, Ang2 promotes the malignant progression of osteosarcoma through various pathways.^[12–14]^

However, the prognostic role of Ang2 in osteosarcoma still remains unclear.^[15–17]^ In this study, the role of Ang2 in the prognosis of patients with osteosarcoma was analyzed using a meta-analysis and The Cancer Genome Atlas (TCGA) database. At the same time, gene set enrichment analysis (GSEA) and immunoosmosis correlation analysis were performed to provide theoretical basis to explore the predictive value and potential mechanism of Ang2 in the prognosis of osteosarcoma.

## 2 Methods

### 2.1 Protocol register

This protocol of systematic review and meta-analysis has been drafted under the guidance of the Preferred Reporting Items for Systematic Reviews and Meta-analyses Protocols. Moreover, it has been registered on open science framework (Registration number: DOI 10.17605/OSF.IO/GWQ53).

### 2.2 Ethics

Since this is a protocol without patient recruitment and personal information collection, the approval of the ethics committee is not required.

### 2.3 Inclusion criteria

(1) Studies exploring the correlation between Ang2 expression and the prognosis of patients with osteosarcoma;
(2) Studies in which patients were divided into two groups according to their expression level of Ang2;
(3) Studies that provided available hazard ratios and 95% confidence intervals of overall survival or survival curves for calculating.

The following studies were excluded:

(1) Reviews, case reports, letters, conference papers, and abstracts;
(2) Cell and animal-related studies.

### 2.4 Search strategy

A systematic literature search of PubMed, Web of Science, China National Knowledge Infrastructure, China Biology Medicine disc, and Wan Fang Data was conducted to identify all relevant articles without language and publication year limitations. The ending date of literature collection was July 2021. The search strategy for PubMed is shown in Table 1.

**Table 1:** Search strategy in PubMed database.

Number	Search terms
#1	Osteosarcoma[MeSH]
#2	Sarcoma, Osteogenic[Title/Abstract]
#3	Osteogenic Sarcoma[Title/Abstract]
#4	Osteosarcoma Tumor[Title/Abstract]
#5	Osteogenic Sarcomas[Title/Abstract]
#6	Osteosarcoma Tumors[Title/Abstract]
#7	Osteosarcomas[Title/Abstract]
#8	Sarcomas, Osteogenic[Title/Abstract]
#9	Tumor, Osteosarcoma[Title/Abstract]
#10	Tumors, Osteosarcoma[Title/Abstract]
#11	or/1–10
#12	Angipoietin-2[Title/Abstract]
#13	Agn2[Title/Abstract]
#14	or/12–13
#15	Prognos^∗^[Title/Abstract]
#16	Survival [Title/Abstract]
#17	Kaplan-Meier [Title/Abstract]
#18	Cox[Title/Abstract]
#19	Univariate [Title/Abstract]
#20	Multivariate[Title/Abstract]
#21	or/15–20
#22	#11 and #14 and #21

### 2.5 Data screening and extraction

**Figure 1:**
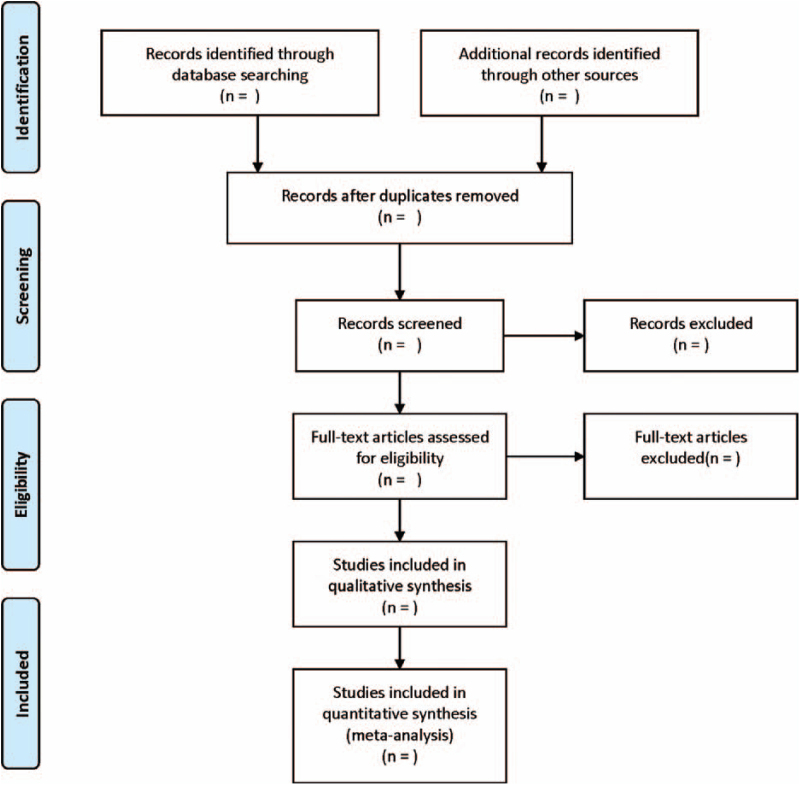
Flow diagram of study selection process.

According to the exclusion and inclusion criteria, 2 investigators independently assess the eligibility of all retrieved papers. Differences between the 2 investigators are resolved through discussion with a third investigator, until consensus is reached. The data extracted from the literature include: first of author, publication date, the country of publication, age, sex, the number of cases, Ang2 assessment methods, detection methods, cut-off value, prognostic indicators, and so on. The literature screening process is shown in Fig. 1.

### 2.6 Literature quality assessment

The Newcastle-Ottawa Scale was used to evaluate the quality of all the published papers.^[18,19]^ If the Newcastle-Ottawa Scale score of the literature is ≥6, it can be considered as high quality.

### 2.7 Dealing with missing data

If there exist insufficient or missing data in the literature, we would only analyze the currently available data and discuss its potential value.

### 2.8 Statistical analysis

#### 2.8.1 Data analysis and processing

STATA 16.0 (STATA Corporation, College Station, TX) was used in this meta-analysis to pool the hazard ratio and its 95% confidence interval. Heterogeneity in included studies was assessed using Cochran *Q* test and Higgins *I*^2^. If the heterogeneity was substantial (*I*^*2*^ > 50%, *P* < .05), the random effect model would be adopted, otherwise the fixed effect model would be used.

#### 2.8.2 Subgroup analysis

Subgroup analysis will be conducted based on the cut-off value of Ang2, survival data sources, and ethnicity.

#### 2.8.3 Sensitivity analysis

A one-by-one elimination method will be adopted for sensitivity analysis.

#### 2.8.4 Publication bias

Publication bias was measured by conducting Begg and Egger tests.^[20]^

## 3 Bioinformatics analysis

### 3.1 TCGA data collection

To further assess the function of Ang2 in osteosarcoma, relevant data from the TCGA database (https://cancergenome.nih.gov/) were collected. Through R software (V 4.1.0 version) and Perl software (V 5.34.0 version), the above downloaded data were summarized. Kaplan–Meier survival analysis, univariate Cox analysis, multivariate Cox analysis, expression difference analysis, and clinical correlation analysis were used to evaluate the role of Ang2 in the prognosis of patients suffering from osteosarcoma. The statistical analysis was performed on R software (V 4.1.0 version), and *P* < .05 indicates significance.

### 3.2 Gene set enrichment analysis

GSEA was used to study the mechanism of Ang2 in osteosarcoma. All data from the gene set database provided by MSIGDB (http://www.gsea-msigdb.org/gsea/msigdb) were analyzed using GSEA4.1 software. According to the median expression of Ang2, osteosarcoma samples were divided into 2 groups, namely, the group with low Ang2 expression and the group with high Ang2 expression. The absolute value of normalized enrichment score (NES) is ≥1.0, *P* < .05 and *q* < 0.25 are considered to be statistically significant on some genomes.

### 3.3 Immunoinfiltration analysis

TIMER (http://cistrome.dfci.harvard.edu/TIMER/) was used to investigate the correlation between Ang2 expression and immune invasion in osteosarcoma. Partial Spearman method with purity correction was used to display the correlation. *P* < .05 indicates that the differences are statistically significant.

## 4 Discussion

The expression of Ang2 is related to the degree of malignancy, lymph node metastasis and distant metastasis of gastric cancer, head and neck squamous cell carcinoma, breast cancer, and lung cancer,^[21–24]^ which suggests that the expression of Ang2 is closely related to tumor. Related studies have exhibited that the prevention of the tumor microenvironment from stimulating the transduction of Ang2 signals can inhibit the metastasis of tumor cells.^[12]^ Ang2 can promote the proliferation of osteosarcoma cells, metastasis of osteosarcoma cells, angiogenesis, and glycolysis.^[25]^ Therefore, Ang2 plays a dual role in tumor progression and anti-tumor immunity. However, the role of Ang2 in the prognosis of patients with osteosarcoma still remains controversial. This study will reveal the prognostic value and mechanism of Ang2 in osteosarcoma through meta-analysis and bioinformatics analysis.

## Author contributions

**Conceptualization:** Ye Qu, Lizhu Liu.

**Data curation:** Xinbo Zhang, Lizhu Liu.

**Formal analysis:** Xinbo Zhang, Ye Qu.

**Funding acquisition:** Ye Qu.

**Investigation:** Xinbo Zhang.

**Methodology:** Xinbo Zhang, Chaoyi Li.

**Project administration:** Ye Qu.

**Software:** Chaoyi Li, Lizhu Liu.

**Supervision:** Ye Qu.

**Validation:** Chaoyi Li, Xinbo Zhang.

**Visualization:** Chaoyi Li, Lizhu Liu.

**Writing – original draft:** Ye Qu, Lizhu Liu.

**Writing – review & editing:** Ye Qu, Lizhu Liu.
